# Methyl (*Z*)-2-chloro-3-(2-methoxy­carbonyl­phen­yl)prop-2-enoate

**DOI:** 10.1107/S160053681000084X

**Published:** 2010-01-16

**Authors:** Casey J. Fadgen, Thomas L. Groy, Seth D. Rose

**Affiliations:** a Department of Chemistry and Biochemistry, Arizona State University, Tempe, Arizona 85287-1604, USA

## Abstract

In the title compound, C_12_H_11_ClO_4_, the propenoate C=C bond is in the *Z* configuration. The propenoate C=O and C=C groups are essentially coplanar [C=C—C=O torsion angle = 172.4 (3)°] with the O atom synperiplanar to the Cl atom. However, the π systems of the aromatic ring and chloro­propenoate substituent are not coplanar; the corresponding dihedral angle is 51.5 (1)°. The noncoplanarity is likely due to steric inter­actions between the propenoate H atom and the *ortho*-methoxy­carbonyl group on the aromatic ring. Even in the observed noncoplanar conformation, the *ortho* C=O to H distance (2.40 Å) is less than the sum of the van der Waals radii of O and H (2.65 Å).

## Related literature

For the structure of 2-nitro­cinnamic acid, in which the alkene group is noncoplanar with the aromatic ring, as in the title compound, see: Smith *et al.* (2006 ▶). For numerous structures of cinnamic acid derivatives, with coplanar aromatic and alkene groups, see: ethyl *p*-methoxy­cinnamate (Luger *et al.*, 1996 ▶), *p*-cresyl cinnamate (Kaitner & Stilinović, 2007 ▶), 4-methyl­coumarin ester of *trans*-cinnamic acid (Yang *et al.*, 2006 ▶), methyl esters of *m*- and *p*-bromo­cinnamic acids (Leiserowitz & Schmidt, 1965 ▶), methyl 3,5-dinitro-*trans*-cinnamate (Sharma *et al.*, 1995 ▶), *N*-cinnamoylsaccharin (Ersanlı *et al.*, 2005 ▶), and 2-ethoxy­cinnamic acid (Fernandes *et al.*, 2001 ▶). For the chlorination step of the synthesis, see: Markó *et al.* (1997 ▶). For van der Waals radii, see: Rowland & Taylor (1996 ▶).
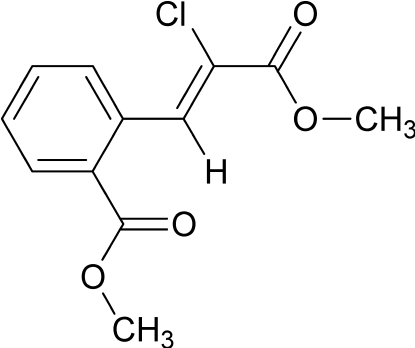


## Experimental

### 

#### Crystal data

C_12_H_11_ClO_4_
*M* *_r_* = 254.66
Monoclinic, 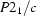

*a* = 10.722 (5) Å
*b* = 15.331 (7) Å
*c* = 7.676 (4) Å
β = 110.395 (10)°
*V* = 1182.7 (10) Å^3^
*Z* = 4
Mo *K*α radiation
μ = 0.32 mm^−1^
*T* = 299 K
0.40 × 0.10 × 0.04 mm

#### Data collection

Bruker SMART APEX CCD diffractometer
Absorption correction: multi-scan (*SADABS*; Bruker, 2008 ▶) *T*_min_ = 0.882, *T*_max_ = 0.988
9313 measured reflections
2110 independent reflections
1559 reflections with *I* > 2σ(*I*)
*R*_int_ = 0.036

#### Refinement

*R*[*F* ^2^ > 2σ(*F* ^2^)] = 0.038
*wR*(*F* ^2^) = 0.096
*S* = 1.01
2110 reflections
156 parameters
H-atom parameters constrained
Δρ_max_ = 0.21 e Å^−3^
Δρ_min_ = −0.18 e Å^−3^

### 

Data collection: *APEX2* and *BIS* (Bruker, 2008 ▶); cell refinement: *SAINT* (Bruker, 2008 ▶); data reduction: *SAINT*; program(s) used to solve structure: *SHELXS97* (Sheldrick, 2008 ▶); program(s) used to refine structure: *SHELXL97* (Sheldrick, 2008 ▶); molecular graphics: *XSHELL* (Bruker, 2004 ▶); software used to prepare material for publication: *SHELXL97*.

## Supplementary Material

Crystal structure: contains datablocks global, I. DOI: 10.1107/S160053681000084X/ya2113sup1.cif

Structure factors: contains datablocks I. DOI: 10.1107/S160053681000084X/ya2113Isup2.hkl

Additional supplementary materials:  crystallographic information; 3D view; checkCIF report

## Figures and Tables

**Fig. 1.**
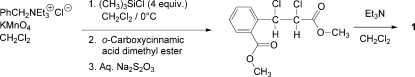
Outline of the synthesis of 1. See Markó et al. (1997) for details.

**Fig. 2.**
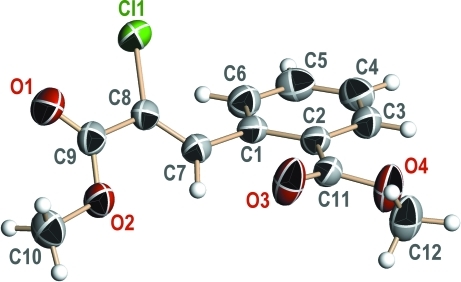
Molecular structure of 1 illustrating the lack of coplanarity of the aromatic ring and chloropropenoate substituent. Thermal displacement ellipsoids are drawn at the 50% probability level.

## supplementary crystallographic information

### Comment

The title compound (1) is of interest in the development of anticancer enzyme inhibitors. Determination of the *E*/*Z* stereochemistry and the preferred conformation were needed for *in silico* docking of 1 and its derivatives to target enzymes.

Synthesis of 1 was carried out as shown in Fig. 1. Esterification of *o*-carboxycinnamic acid was accomplished by treatment with CH_3_OH/HCl. The subsequent formation of the *Z*-isomer during synthesis can be rationalized on the basis of the known lack of stereospecificity of the dichlorination reaction when applied to aromatic-substituted alkenes, carried out essentially by the method of Markó *et al.* (1997).

Subsequent elimination of HCl from the dichloro derivative by treatment with triethylamine in dichloromethane produced 1 in ~10:1 ratio (by NMR) with its *E* isomer. Regioselectivity (*i.e.*, preferential formation of the 2-chloropropenoate over 3-chloropropenoate) results from the more facile deprotonation of the more acidic H atom in the α-position to the ester carbonyl group, with loss of the 3-chlorine atom.

Fig. 2 shows that 1 has *Z* configuration at the alkene double bond and that the π systems of the aromatic ring and the chloropropenoate substituent are not coplanar. The C2—C1—C7—C8 torsion angle is 133.5 (3)° and the C6—C1—C7—C8 torsion angle is -49.4 (4)°; the dihedral angle formed by the plane of the aromatic ring and the plane of the chloropropenoate substituent (C7, C8, C9, Cl1, O1, O2) is equal to 51.5 (1)°. The *ortho*-methoxycarbonyl substituent is slightly non-coplanar with the aromatic ring, as shown by the O3—C11—C2—C1 torsion angle of -10.7 (4)°. Loss of resonance stabilization by twisting of the *ortho*-methoxycarbonyl and propenoate groups relative to the aromatic ring is presumably balanced by relief of steric strain due to proximity of the *ortho*-carbonyl oxygen and the propenoate hydrogen.

### Experimental

2-Carboxycinnamic acid (2-CCA) dimethyl ester.

Acetyl chloride (1 ml) was slowly added to 25 ml of cold methanol in a 100-ml round-bottom flask. The reaction mixture was warmed slowly then refluxed for 10 minutes prior to addition of 381 mg (2 mmol) of 2-CCA. The reaction mixture was refluxed and monitored by thin-layer chromatography (TLC). Typically, the reaction was complete in less than 2 h. The reaction mixture was concentrated by rotary evaporation and passed through a short silica gel column with dichloromethane elution to yield 420 mg (~95%) of a viscous, clear oil.

Dichlorination of 2-CCA dimethyl ester.

KMnO_4_ (730 mg, 4.62 mmol) and benzyltriethylammonium chloride (1.040 g, 4.57 mmol) were stirred in 20 ml of dry dichloromethane. The resulting dark purple solution was cooled on ice for 20 min. Chlorotrimethylsilane (~2 g, 18.4 mmol) was slowly added to the stirred solution, which gradually took on a dark green color. The reaction mixture was allowed to warm to room temperature, and 2-CCA dimethyl ester (1 g, 4.55 mmol) was added. The reaction mixture was monitored by TLC, but the reaction was terminated prior to completion because of reagent degradation (green color vanishing and dark or brown color appearing, along with multiple spots appearing upon TLC). The organic solution was successively washed with 0.1 *M* sodium thiosulfate and brine, followed by drying over anhydrous sodium sulfate. A silica gel column was used to separate the mixture (elution with 1:1 hexanes–chloroform). Recrystallization from dichloromethane/hexanes yielded 640 mg of white solid. *M*.p. 70.5–72 °C. ^1^H NMR (400 MHz in CDCl_3_) δ, 7.97 (d, 1H, J = 8.0 Hz), 7.69 (d, 1H, J = 7.6 Hz), 7.60 (t, 1H, J = 7.6 Hz), 7.44 (app t, 1H, J = 7.6, 8.0 Hz), 6.62 (d, 1H, J = 10.4 Hz), 4.75 (d, 1H, 10.4 Hz), 3.95 (s, 3H), 3.88 (s, 3H); ^13^C NMR (100 MHz in CDCl_3_) δ, 167.5, 167.0, 137.7, 132.6, 130.6, 130.0, 128.9, 128.6, 59.2, 55.8, 53.4, 52.6. Anal. Calcd (%) for C_12_H_11_ClO_4_: C, 49.51; H, 4.15. Found: C, 49.30; H, 4.11.

Elimination of HCl to form 1.

In 2 ml of dichloromethane was dissolved 300 mg (1.03 mmol) of the above dichloro ester. Triethylamine (110 mg, 1.10 mmol) was then added, and the reaction was monitored by TLC. The reaction mixture was washed successively with ~5 ml of 1.0 *M* HCl and brine, followed by drying over anhydrous sodium sulfate. Formation of an approximately 10:1 ratio of 1 to its *E* isomer was indicated by ^1^H NMR spectroscopy of the crude product. Subsequent crystallizations from dichloromethane/hexanes yielded ~90 mg of white solid, 1 (0.35 mmol, 34%). ^1^H NMR (500 MHz in CDCl_3_) δ, 8.45 (s, 1H), 8.05 (d, 1H, J = 12.5 Hz), 7.66 (d, 1H, J = 8 Hz), 7.57 (app t, 1H, J = 12.5, 8 Hz), 7.44 (app t, 1H, J = 8, 7.5 Hz), 3.90 (s, 3H), 3.88 (s, 3H).

### Refinement

All H-atoms were placed geometrically (C—H 0.93 and 0.96 Å for aromatic and methyl H atoms respectively) and included in the refinement in the riding motion approximation with *U*_iso_(H) = 1.2*U*_eq_(C) for CH and *U*_iso_(H) = 1.5*U*_eq_(C) for CH_3_.

### Crystal data

**Table:**

C_12_H_11_ClO_4_	*F*(000) = 528
*M**_r_* = 254.66	*D*_x_ = 1.430 Mg m^−^^3^
Monoclinic, *P*2_1_/*c*	Mo *K*α radiation, λ = 0.71073 Å
Hall symbol: -P 2ybc	Cell parameters from 536 reflections
*a* = 10.722 (5) Å	θ = 2.4–21.4°
*b* = 15.331 (7) Å	µ = 0.32 mm^−^^1^
*c* = 7.676 (4) Å	*T* = 299 K
β = 110.395 (10)°	Needle, colourless
*V* = 1182.7 (10) Å^3^	0.40 × 0.10 × 0.04 mm
*Z* = 4	

### Data collection

**Table:**

Bruker SMART APEX CCD diffractometer	2110 independent reflections
Radiation source: sealed tube	1559 reflections with *I* > 2σ(*I*)
graphite	*R*_int_ = 0.036
Detector resolution: 8.3330 pixels mm^-1^	θ_max_ = 25.2°, θ_min_ = 2.0°
ω and φ scans	*h* = −12→12
Absorption correction: multi-scan (*SADABS*; Bruker, 2008)	*k* = −18→18
*T*_min_ = 0.882, *T*_max_ = 0.988	*l* = −8→9
9313 measured reflections	

### Refinement

**Table:**

Refinement on *F*^2^	Primary atom site location: structure-invariant direct methods
Least-squares matrix: full	Secondary atom site location: difference Fourier map
*R*[*F*^2^ > 2σ(*F*^2^)] = 0.038	Hydrogen site location: inferred from neighbouring sites
*wR*(*F*^2^) = 0.096	H-atom parameters constrained
*S* = 1.01	*w* = 1/[σ^2^(*F*_o_^2^) + (0.0409*P*)^2^ + 0.4245*P*] where *P* = (*F*_o_^2^ + 2*F*_c_^2^)/3
2110 reflections	(Δ/σ)_max_ < 0.001
156 parameters	Δρ_max_ = 0.21 e Å^−^^3^
0 restraints	Δρ_min_ = −0.18 e Å^−^^3^

### Special details

**Table:**

Geometry. All e.s.d.'s (except the e.s.d. in the dihedral angle between two l.s. planes) are estimated using the full covariance matrix. The cell e.s.d.'s are taken into account individually in the estimation of e.s.d.'s in distances, angles and torsion angles; correlations between e.s.d.'s in cell parameters are only used when they are defined by crystal symmetry. An approximate (isotropic) treatment of cell e.s.d.'s is used for estimating e.s.d.'s involving l.s. planes.
Refinement. Refinement of *F*^2^ against ALL reflections. The weighted *R*-factor *wR* and goodness of fit *S* are based on *F*^2^, conventional *R*-factors *R* are based on *F*, with *F* set to zero for negative *F*^2^. The threshold expression of *F*^2^ > σ(*F*^2^) is used only for calculating *R*-factors(gt) *etc*. and is not relevant to the choice of reflections for refinement. *R*-factors based on *F*^2^ are statistically about twice as large as those based on *F*, and *R*- factors based on ALL data will be even larger.

### Fractional atomic coordinates and isotropic or equivalent isotropic displacement parameters (Å^2^)

**Table:**

	*x*	*y*	*z*	*U*_iso_*/*U*_eq_	
Cl1	−0.00350 (6)	0.17184 (4)	0.01458 (9)	0.0599 (2)	
O1	−0.01434 (17)	0.36083 (12)	0.0476 (2)	0.0699 (5)	
O2	0.18915 (14)	0.37829 (10)	0.2585 (2)	0.0550 (4)	
O3	0.46537 (17)	0.23575 (11)	0.1480 (2)	0.0734 (5)	
O4	0.61609 (16)	0.13201 (11)	0.2032 (2)	0.0697 (5)	
C1	0.2933 (2)	0.11550 (13)	0.2545 (2)	0.0420 (5)	
C2	0.4135 (2)	0.09334 (12)	0.2308 (2)	0.0418 (5)	
C3	0.4545 (2)	0.00641 (13)	0.2476 (2)	0.0493 (5)	
H3	0.5336	−0.0081	0.2304	0.059*	
C4	0.3807 (2)	−0.05806 (14)	0.2890 (2)	0.0567 (5)	
H4	0.4092	−0.1157	0.2984	0.068*	
C5	0.2641 (2)	−0.03686 (16)	0.3165 (2)	0.0569 (5)	
H5	0.2143	−0.0801	0.347	0.068*	
C6	0.2214 (2)	0.04850 (16)	0.2986 (2)	0.0516 (5)	
H6	0.1421	0.0619	0.3166	0.062*	
C7	0.2465 (2)	0.20628 (13)	0.2425 (2)	0.0442 (5)	
H7	0.3094	0.2473	0.3075	0.053*	
C8	0.1264 (2)	0.23667 (13)	0.1511 (2)	0.0435 (5)	
C9	0.0900 (2)	0.33090 (16)	0.1439 (2)	0.0474 (5)	
C10	0.1623 (2)	0.46999 (14)	0.2694 (4)	0.0634 (7)	
H10A	0.1366	0.4954	0.1476	0.095*	
H10B	0.2409	0.4985	0.3501	0.095*	
H10C	0.0915	0.4771	0.3177	0.095*	
C11	0.4975 (2)	0.16172 (14)	0.1891 (2)	0.0445 (5)	
C12	0.7057 (2)	0.19250 (18)	0.1647 (5)	0.0712 (8)	
H12A	0.6621	0.2196	0.0463	0.107*	
H12B	0.7832	0.1619	0.1624	0.107*	
H12C	0.7317	0.2364	0.2598	0.107*	

### Atomic displacement parameters (Å^2^)

**Table:**

	*U*^11^	*U*^22^	*U*^33^	*U*^12^	*U*^13^	*U*^23^
Cl1	0.0400 (2)	0.0651 (4)	0.0684 (4)	−0.0037 (2)	0.0109 (2)	−0.0123 (2)
O1	0.0565 (11)	0.0651 (11)	0.0728 (13)	0.0194 (9)	0.0032 (9)	0.0021 (9)
O2	0.0482 (9)	0.0406 (9)	0.0716 (11)	0.0052 (7)	0.0150 (8)	−0.0016 (8)
O3	0.0528 (10)	0.0453 (10)	0.1248 (17)	0.0069 (8)	0.0344 (11)	0.0199 (11)
O4	0.0495 (10)	0.0495 (10)	0.1210 (16)	0.0077 (8)	0.0437 (10)	0.0151 (10)
C1	0.0402 (11)	0.0401 (11)	0.0417 (11)	−0.0016 (9)	0.0092 (10)	−0.0038 (10)
C2	0.0391 (11)	0.0373 (11)	0.0446 (13)	−0.0002 (9)	0.0091 (10)	−0.0013 (10)
C3	0.0444 (13)	0.0423 (13)	0.0576 (14)	0.0029 (10)	0.0131 (11)	−0.0022 (11)
C4	0.0616 (16)	0.0360 (11)	0.0651 (16)	−0.0001 (11)	0.0127 (13)	0.0004 (11)
C5	0.0609 (16)	0.0467 (14)	0.0594 (16)	−0.0116 (11)	0.0164 (13)	0.0031 (11)
C6	0.0464 (14)	0.0527 (15)	0.0553 (15)	−0.0034 (11)	0.0171 (11)	−0.0007 (11)
C7	0.0388 (11)	0.0438 (11)	0.0485 (13)	−0.0016 (10)	0.0133 (10)	−0.0033 (10)
C8	0.0389 (11)	0.0459 (13)	0.0473 (13)	0.0001 (10)	0.0169 (10)	−0.0049 (10)
C9	0.0429 (13)	0.0556 (14)	0.0467 (13)	0.0066 (11)	0.0196 (11)	0.0030 (11)
C10	0.0690 (17)	0.0411 (13)	0.079 (2)	0.0054 (11)	0.0241 (15)	0.0009 (13)
C11	0.0379 (11)	0.0425 (14)	0.0500 (13)	0.0022 (10)	0.0112 (10)	−0.0008 (10)
C12	0.0517 (16)	0.0614 (17)	0.112 (2)	−0.0011 (13)	0.0425 (16)	0.0077 (16)

### Geometric parameters (Å, °)

**Table:**

Cl1—C8	1.734 (2)	C4—C5	1.378 (3)
O1—C9	1.197 (3)	C4—H4	0.93
O2—C9	1.334 (3)	C5—C6	1.377 (3)
O2—C10	1.443 (3)	C5—H5	0.93
O3—C11	1.196 (3)	C6—H6	0.93
O4—C11	1.319 (3)	C7—C8	1.318 (3)
O4—C12	1.439 (3)	C7—H7	0.93
C1—C6	1.396 (3)	C8—C9	1.492 (3)
C1—C2	1.405 (3)	C10—H10A	0.96
C1—C7	1.472 (3)	C10—H10B	0.96
C2—C3	1.395 (3)	C10—H10C	0.96
C2—C11	1.487 (3)	C12—H12A	0.96
C3—C4	1.371 (3)	C12—H12B	0.96
C3—H3	0.93	C12—H12C	0.96
			
C9—O2—C10	116.07 (18)	C1—C7—H7	116.0
C11—O4—C12	117.0 (2)	C7—C8—C9	123.8 (2)
C6—C1—C2	117.6 (2)	C7—C8—Cl1	123.29 (18)
C6—C1—C7	120.4 (2)	C9—C8—Cl1	112.84 (16)
C2—C1—C7	122.0 (2)	O1—C9—O2	123.9 (2)
C3—C2—C1	119.5 (2)	O1—C9—C8	124.7 (2)
C3—C2—C11	119.9 (2)	O2—C9—C8	111.4 (2)
C1—C2—C11	120.6 (2)	O2—C10—H10A	109.5
C4—C3—C2	121.5 (2)	O2—C10—H10B	109.5
C4—C3—H3	119.3	H10A—C10—H10B	109.5
C2—C3—H3	119.3	O2—C10—H10C	109.5
C3—C4—C5	119.6 (2)	H10A—C10—H10C	109.5
C3—C4—H4	120.2	H10B—C10—H10C	109.5
C5—C4—H4	120.2	O3—C11—O4	122.0 (2)
C6—C5—C4	119.8 (2)	O3—C11—C2	125.8 (2)
C6—C5—H5	120.1	O4—C11—C2	112.2 (2)
C4—C5—H5	120.1	O4—C12—H12A	109.5
C5—C6—C1	122.1 (2)	O4—C12—H12B	109.5
C5—C6—H6	119.0	H12A—C12—H12B	109.5
C1—C6—H6	119.0	O4—C12—H12C	109.5
C8—C7—C1	128.1 (2)	H12A—C12—H12C	109.5
C8—C7—H7	116.0	H12B—C12—H12C	109.5
			
C2—C1—C7—C8	133.53 (25)	O3—C11—C2—C1	−10.69 (36)
C6—C1—C7—C8	−49.38 (34)		
